# Forecasted attribution of the human influence on Hurricane Florence

**DOI:** 10.1126/sciadv.aaw9253

**Published:** 2020-01-01

**Authors:** K. A. Reed, A. M. Stansfield, M. F. Wehner, C. M. Zarzycki

**Affiliations:** 1 School of Marine and Atmospheric Sciences, Stony Brook University, Stony Brook, NY, USA.; 2 Lawrence Berkeley National Laboratory, Berkeley, CA, USA.; 3 Climate and Global Dynamics Laboratory, National Center for Atmospheric Research, Boulder, CO, USA.; 4 Department of Meteorology and Atmospheric Science, Pennsylvania State University, State College, PA, USA.

## Abstract

Changes in extreme weather, such as tropical cyclones, are one of the most serious ways society experiences the impact of climate change. Advance forecasted conditional attribution statements, using a numerical model, were made about the anthropogenic climate change influence on an individual tropical cyclone, Hurricane Florence. Mean total overland rainfall amounts associated with the forecasted storm’s core were increased by 4.9 ± 4.6% with local maximum amounts experiencing increases of 3.8 ± 5.7% due to climate change. A slight increase in the forecasted storm size of 1 to 2% was also attributed. This work reviews our forecasted attribution statement with the benefit of hindsight, demonstrating credibility of advance attribution statements for tropical cyclones.

## Teaser

Hurricane Florence produced substantially more extreme rainfall and was spatially larger due to human-induced climate change.

## INTRODUCTION

Change in extreme weather and extreme climate events is a principal way that society experiences the impacts of anthropogenic climate change (*1*). Tropical cyclones, extreme temperature, drought, and severe weather account for most of the increasing damages and economic impacts in the United States (*2*). Since the pioneering analysis of the 2003 European heatwave (*3*), substantial advancements in attribution statements about the influence of anthropogenic climate change on the frequency and magnitude of individual extreme weather and climate events have been made (*4*). While a human influence on the precipitation from individual extreme storms has been identified before (*5*, *6*), Hurricane Harvey was the first true tropical cyclone to undergo these analyses (*7*–*11*). The lag time between extreme weather events and subsequent attribution statements has been steadily decreasing as the community’s expertise has developed to a point that several European weather forecast agencies are currently planning operational attribution capabilities in the near future (*12*). Recently, the “hindcast attribution method” (*13*), described in Methods, was introduced to make attribution statements about present-day tropical cyclones and other severe storms, similar to the “pseudo–global warming” approach for the projection of future climate extremes (*14*, *15*). This attribution methodology has been used to identify the current and future human influence, if any, on the wind speed and precipitation of 15 historical tropical cyclones, including Hurricanes Katrina, Maria, and Irma (*16*), and has been referred to as a “storyline” approach to attribution (*17*).

Extreme event attribution is an exercise in causality. As with any complex phenomena, the genesis of a tropical cyclone occurs when the state of the atmosphere-ocean system is favorable for it (*18*, *19*). The role of anthropogenic climate change on a tropical cyclone’s existence, if any, is manifested through changes in the magnitude or frequency of these causal conditions. Event attribution statements then are statements about the changes in the magnitude or probability of an event. In the hindcast attribution approach, the following conditions are imposed: (i) observed human-caused changes in the composition of the atmosphere, including greenhouse gases and aerosols; (ii) credible estimates of human-induced changes in the ocean surface temperatures and mean atmospheric state aloft; and (iii) that cyclogenesis has occurred under a plausible synoptic environment. Because of these stringent conditions, especially the last one, storyline attribution statements must be considered as incomplete assessments of the effect of anthropogenic climate change on extreme weather events and conditional on the event’s underlying existence.

While both theoretical models of intense tropical cyclones (*20*, *21*) and multidecadal integrations of tropical cyclone permitting climate models suggest that the most intense tropical cyclones become more frequent and more intense in a warmer climate (*22*, *23*), the detection of these changes in the observational record is questionable (*24*, *25*). However, trends in extreme precipitation over all storm types have been detected in global analyses and attributed to human-induced changes to the composition of the atmosphere (*26*, *27*). Three of the aforementioned Hurricane Harvey studies directly found large attributable increases (10 to 38%) on that storm’s precipitation, each using different methodologies. It appears that the human influence on tropical cyclone precipitation is emerging faster than on maximum wind speeds (*16*).

Two days before the landfall of Hurricane Florence (*28*), we publicly forecasted the following attribution statements:

1) Hurricane Florence would be slightly more intense (lower surface pressure) for a longer portion of the forecast period due to climate change.

2) Hurricane Florence rainfall amounts over the Carolinas would be increased by over 50% due to climate change and are linked to warmer sea surface temperatures and available moisture in the atmosphere.

3) Hurricane Florence would be about 80 km larger due to the effect of climate change on the large-scale environment around the storm.

These statements were made on the basis of simulations using the hindcast attribution method but as a forecast in advance of the storm’s landfall. This paper reviews the forecasted attribution with the benefit of hindsight.

## RESULTS

### Forecast attribution framework applied to Hurricane Florence

Florence, a North Atlantic hurricane, made landfall as a category 1 storm on the coast of North Carolina at 11:15Z on 14 September 2018. After landfall, Florence’s forward motion slowed as it dropped large amounts of rain throughout the region before moving farther inland on 17 September 2018. The resulting inland flooding caused extensive damage and hardships for North and South Carolinians.

**Fig. 1:**
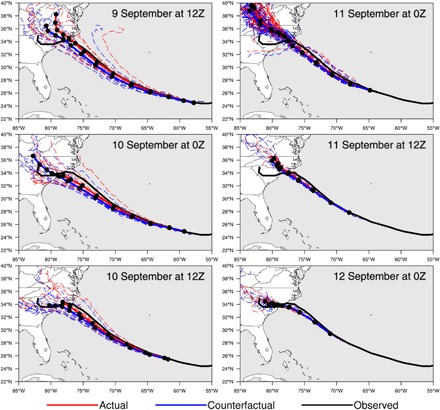
Simulated and observed storm tracks. Model tracks of the 7-day Actual (red) and Counterfactual (blue) forecasts for different initialization times. Solid lines are the ensemble mean, and dashed lines are the individual ensemble members. Black lines are observed track. Black dots on the ensemble mean tracks represent the location of the hurricane center at 12-hour intervals.

A key aspect of any attribution modeling study (*13*, *29*, *30*) is to verify that the models used are fit for the intended purposes. In our approach, the forecasted ensemble simulations in the actual world (here called “Actual”) and a counterfactual world that might have been had humans not altered the atmosphere (here called “Counterfactual”) must reasonably represent the observed storm’s evolution, especially its track. Figure 1 shows the 7-day hindcasted track for Hurricane Florence initialized every 12 hours starting 9 September at 12Z from the Community Atmosphere Model version 5 (CAM5) based on previous CAM5 hindcasts (*31*). Each configuration/initialization pair consists of 10 ensemble members, although the 11 September 00Z forecast period was expanded after the storm to a 100-member ensemble to allow for a more thorough attribution analysis. Our forecasted attribution statement, released to the public on 12 September, was also initialized at that time but using only the original 10 members due to real-time computing constraints. From Fig. 1, most Actual simulations reasonably represent the observed track from the National Hurricane Center (NHC) of Hurricane Florence. Quantitative track error analysis indicates that the CAM5 track error was within the spread of that from operational numerical weather prediction models and the NHC (see Supplementary Materials).

A more critical test of the system’s fitness for purpose is the timing and location of Hurricane Florence’s landfall. All Actual ensemble hindcasts, except for the 11 September 12Z ensemble, simulated a median landfall time within 4 hours of the observed landfall of Hurricane Florence. Focusing on the Actual 11 September 00Z ensemble, the slowdown of Hurricane Florence near landfall and the landfall timing is well captured by the model, with a median landfall time of 14 September 15Z. In addition, Fig. 1 shows that the model captures the location of Hurricane Florence’s landfall on 14 September, with 65 members of the 11 September 00Z ensemble predicting a landfall location within about 30 km of the observed location and 96 of these ensemble members within 200 km. Given that the timing and location of landfall were reasonably simulated, both of which are important for overland rainfall, we conclude that these 96 members of the Actual ensemble are fit for purpose in simulating the observed storm. The comparison of the Actual ensemble to rainfall and dynamical measures of intensity are discussed later in the analysis.

To represent the storm in a world without anthropogenic climate change, we performed a suite of Counterfactual ensemble hindcasts as per the Actual ensemble but with the large-scale climate change signal removed from the initial and boundary conditions. As in previous studies, this was performed by subtracting the attributable warming from the sea surface temperature (about 0.75°C near the Carolina coast as determined from the Coupled Model Intercomparison Project Phase 5 ensemble) and modifying atmospheric temperature and moisture (*5*, *16*). Details of these modifications are discussed in the Supplementary Materials. Comparing the Actual to Counterfactual ensembles in Fig. 1, there is little difference in the simulated storm track and landfall location between the two ensembles. The translational speed of the storm center is also not affected in the Counterfactual simulations. While recent work has identified a slowing of average tropical cyclone translational speed due to climate change–induced circulation changes (*32*), this is not explicitly imposed within this modeling framework due to the conditional nature of the initialization, particularly the synoptic-scale forcing. Because of this, 34 of the Counterfactual 11 September 00Z ensemble members predicted a landfall location within 30 km of the observed location and 96 members within 200 km.

This additional similarity in tracks and landfall timing between the Actual and Counterfactual 11 September 00Z ensemble simulations and their agreement with the observed storm leads us to conclude that this system is fit-for-purpose for a conditional attribution. For the initial 10-member ensembles performed for our forecasted attribution, we reached this same conclusion based on the agreement between our advance simulations and the official NHC forecast before a public statement in advance of landfall was made.

### Climate change impact on extreme rainfall

**Fig. 2:**
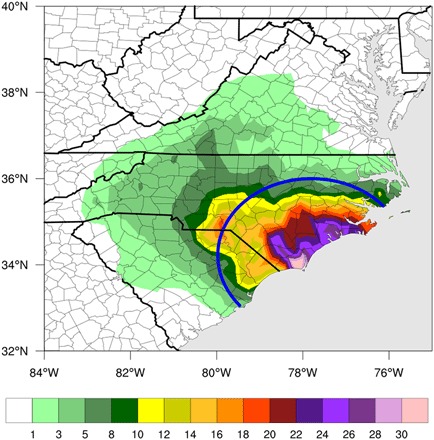
Observed Florence rainfall. Total observed accumulated precipitation (inches) within 500 km of Hurricane Florence’s landfall location. NWS observations are regridded onto the CAM5 grid. Blue line represents 200 km around the location of landfall used for rainfall analysis.

The National Weather Service (NWS) rainfall gridded observational dataset estimates locations near the landfall location of Hurricane Florence experienced over 30 inches (762 mm) of rain, with the maximum rainfall total of 32.9 inches (836 mm) near Wilmington, NC (Fig. 2). All analysis performed for the remainder of this study focuses on the 11 September at 00Z ensembles and the 96 members that make landfall within 200 km of the observed landfall of Hurricane Florence. Analysis of the accumulated rainfall overland associated with the Actual ensemble initialized on 11 September at 00Z demonstrates that the framework well simulates amounts of observed rainfall (fig. S2). While the precise spatial distribution of rainfall is dependent on the storm tracks of each individual realization, accumulated rainfall amounts in excess of 30 inches are simulated in many of the Actual ensembles. The examination of the Counterfactual ensemble (fig. S3) exhibits that the accumulated rainfall associated with the landfalling storms is noticeably reduced, although rainfall amounts in excess of 30 inches are still possible in the cooler Counterfactual world, again with variations in spatial distribution depending on simulated storm track and landfall.

**Fig. 3:**
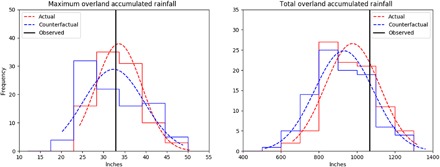
Simulated changes in storm rainfall. Histograms of the (left) maximum accumulated rainfall amounts and (right) total accumulated rainfall within 200 km and 48 hours of the model landfall for the Actual (red) and Counterfactual (blue) 11 September 00Z ensembles. Dashed lines are Gaussian fits to the data. Only the 96 ensemble members that make landfall within 200 km of the observed landfall location are included. The NWS observations are marked with vertical black lines.

We focus on a region of approximately 200 km around the simulated storm’s landfall for each individual ensemble member, as this is the core of the storm and the location of the heaviest precipitation. Figure 3 (left) shows the distribution of the maximum accumulated rainfall amount at any grid point within 200 km of the forecasted landfalls for the 96 Actual and Counterfactual ensemble members that make landfall within 200 km of the observed landfall, with a slight shift toward higher maximum values of rainfall in the Actual simulation. The mean value of maximum accumulated precipitation in the Actual ensemble is 33.6 inches (853 mm), comparable with the observed value of 32.9 inches (836 mm) but about 4% larger than the mean value of 32.4 inches (823 mm) in the Counterfactual ensemble. When comparing median values, the Actual ensemble maximum accumulated precipitation is nearly 5% larger than the Counterfactual ensemble without the anthropogenic signal. When focusing on all grid points within 200 km of landfall, 2.9% of those points have an accumulated rainfall of 30 inches or more in the Actual ensemble, an increase from 2.7% in the Counterfactual ensemble (over a 7% increase in points with over 30 inches of rainfall).

The total accumulated overland precipitation over all grid points within 200 km of the individual ensemble landfall location and within 48 hours of individual landfall time (Fig. 3, right) in the Actual ensemble also shows a shift toward the higher values of rainfall compared to the Counterfactual ensemble. The mean value of total rainfall in the Actual ensemble is 977.0 inches (24,816 mm), nearly 5% larger than the mean value of 931.7 inches (23,665 mm) in the Counterfactual ensemble. The median of the total overland precipitation in the Actual ensemble is 955.1 inches (24,260 mm) compared with 909.9 inches (23,111 mm) in the Counterfactual ensemble, a 5% increase. Note that, again, the Actual ensemble compares reasonably well with the observed total accumulated overland rainfall within 200 km and 48 hours of landfall of 1066.7 inches (27,094 mm). However, for longer temporal windows after landfall (i.e., 72 hours), the Actual ensemble underestimates the total rainfall within 200 km due to differences in the simulated track and storm’s translation speed after landfall compared to the observed (Fig. 1). The differences between the two ensembles is statistically significant at the 80% confidence level for the local maximum precipitation and the 95% confidence level for the total overland precipitation near the core of the storm for the ensembles using either a one-sided or two-sided Student’s *t* test.

Our forecasted attribution statement of 12 September 2018 “increased by over 50%” was based on only 10 members of the original 11 September 00Z ensemble and did not account for slight differences in track, an amount outside the 95% confidence interval of the full 96-member hindcasted ensemble (Table 1). We investigated the sensitivity of a smaller ensemble using 10-member subsets derived from the full ensemble using a bootstrap analysis of 1000 samples, finding that the 95% confidence interval for difference in the overland precipitation ranged from −8.2 to 21.4% (Table 1).

**Table 1:** Summary attributable changes in rainfall. Comparison of 11 September 00Z full ensembles and 10-member ensemble subsets to the forecasted attribution statement. For the full ensembles, differences in mean values are provided with a 95% confidence interval, which given the large ensemble size matches the 95% confidence interval derived from a bootstrap analysis of 1000 samples. The 95% confidence interval for 10-member ensemble subsets is also derived from a bootstrap analysis of 1000 samples.

	**Difference in** **mean** **maximum** **precipitation**	**Difference in** **mean total** **overland** **precipitation**	**Difference in** **mean** **maximum** **outer storm** **diameter**
**Full ensemble** Mean with 95% confidence interval (conventional)	3.8 ± 5.7%	4.9 ± 4.6%	9.1 ± 6.1 km
**Full ensemble** 95% confidence interval (bootstrapped)	−1.2 to 9.6%	0.7 to 10.3%	3.1 to 15.3 km
**10-member** **ensembles** **subsets** 95% confidence interval (bootstrapped)	−10.8 to 22.5%	−8.2 to 21.4%	−8.6 to 28.5 km
Forecast	—	50%	80 km

### Attribution of storm size

**Fig. 4:**
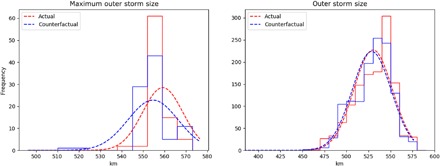
Simulated changes in storm size. Histograms of (left) maximum and (right) all 3-hour radii of the azimuthal-mean 8 m/s azimuthal wind for the Actual (red) and Counterfactual (blue) 11 September 00Z ensembles during the 36-hour period (12 September 00Z to 13 September 12Z). This time period is selected because it begins 24 hours after initialization to allow for model spin-up and ends 24 hours before ensemble median landfall to ensure that the storm size is not affected by interactions with land. Dashed lines are Gaussian fits to the data. Only the 96 ensemble members that make landfall within 200 km of the observed landfall location are included.

Hurricane size is also understood to be an important factor in storm damage potential (*33*–*35*). Figure 4 (left) shows the distribution of the maximum forecasted outer radius of Hurricane Florence (here estimated as the radius at wind speeds of 8 m/s) (*36*) during a 36-hour period well before landfall for the Actual and Counterfactual 11 September 00Z ensembles for the storms that make landfall within 200 km of the observed. The distribution of the Actual ensemble is shifted toward larger storm sizes compared with the Counterfactual ensemble in which the large-scale climate change signal is removed. In particular, the Actual ensemble predicted a storm that is 4.5 km in mean radius or more than 9.1 km in mean diameter, larger than the ensemble without climate change. This represents a 1.6% increase in the area of the mean forecasted storm due to climate change. When all times are considered during the 36-hour period, as shown in Fig. 4 (right), the shift in outer storm size is not as apparent, with less than 1% increase in the mean area in the Actual ensemble. While the change in maximum outer storm size is substantially smaller than that stated in the 12 September forecasted attribution release (see Table 1), there are instances when the difference in median diameter between the ensembles is considerably larger depending on the ensemble size (Table 1). Again, the differences stated in Table 1 are statistically significant at over a 99% confidence level using either a one-sided or two-sided Student’s *t* test.

This attributed increase in size is not obvious from changes in intensity, as estimated by instantaneous maximum near-surface wind speeds (fig. S4), which is less than 2% between the ensembles, consistent with previous attribution work (*16*). Intensity, as determined by minimum surface pressure in the center of storm (fig. S4), has been shown to depend on the maximum near-surface wind speeds, outer storm size, and latitude (*36*). Given that both the Actual and Counterfactual ensembles have similar storm tracks, and therefore latitudes, the attributed changes in outer storms size (Fig. 4) and the smaller change in maximum near-surface wind speeds corroborate the attributable increase (~4%) in the forecasted minimum surface pressure deficit (fig. S4) in the Actual ensemble given a similar background environmental surface pressure. However, a more detailed investigation of storm structure changes, which may be linked to the rainfall difference shown here, is needed.

## DISCUSSION

This work presents and evaluates the first documented forecast attribution study performed in advance of a landfalling hurricane. The extreme rainfall amounts of Hurricane Florence are simulated to be significantly increased because of human-induced climate change. Our forecasted attribution statement based on the original 10-ensemble members for Hurricane Florence was made 2 days before landfall (*28*) compared with additional ensemble simulations completed after the storm is presented here. This analysis quantifies confidence in the forecast attribution statement made in advance of the storm. Similar to previous work with other tropical cyclones (*16*), the human influence on Florence is most apparent in precipitation rather than maximum instantaneous wind speed. The extreme rainfall was increased by up to 10%, and the fraction of rainfall accumulations of more than 30 inches was increased by more than 7% of what it would have been without climate change. This result is on the lower end of the 10 to 38% increase in storm total precipitation found for a similar slow-moving storm, Hurricane Harvey (*7*–*9*, *11*). When compared to these estimated precipitation changes for Hurricane Harvey, those presented here for Hurricane Florence not only are more in agreement with the simple thermodynamic scaling arguments as determined by Clausius-Clapeyron scaling (~7% for Harvey and ~5% for Florence) but are also consistent with the range of changes simulated in several other intense storms (*16*, *37*). To test whether selection bias plays a notable role in the attributed signal discussed in the analysis, we completed an additional 10-member ensemble suite in which a climate change signal was added to the Actual ensemble. This test results in a further increase in maximum rainfall, suggesting that selection bias did not contribute substantially to the results.

Before landfall, we forecasted that Hurricane Florence was about 80 km larger in diameter due to climate change. With the additional simulations, this statement is modified to the maximum size of Hurricane Florence, which is about 9 km larger in mean maximum diameter (or a 1.6% increase in storm area) due to climate change. However, instances in which the simulated storm is larger in diameter within the ensemble are still shown to be possible but is dependent on ensemble size. We also made a qualitative statement that the storm would be slightly more intense for a longer portion of the forecast period due to climate change, as measured by minimum surface pressure. While this conclusion holds in the post-storm ensembles, this remains the most uncertain finding of this work. While our ensemble intensities for Hurricane Florence are in line with NHC forecasts, simulated hurricane intensity (particularly surface wind) can be deficient in global modeling frameworks (*31*, *38*).

Hindcasting offers a luxury of time not afforded to forecasting. The quantitative aspects of our forecasted attribution statements fall outside broad confidence intervals of our hindcasted statements and are quite different from the hindcasted best estimates. This discrepancy is, in part, due to the discovery during the refining of the hindcast framework that the forecasted Counterfactual ensemble incorrectly set the lower boundary condition for sea surface temperatures while correctly setting the initial conditions, having the effect of increasing the climate change signal by 1° to 3° off the coast of North Carolina. Furthermore, limits in human and computational resources limited our forecasted ensemble sizes to just 10 members, not enough to construct defensible confidence intervals. An important lesson learned from this exercise is that variability across tropical cyclone simulations is large enough to cause uncertainties in the attributable effect of climate change on storm characteristics. The design of the numerical approach of an attribution study must take several important factors into account, all of which affect the demands on available human and computational resources. These factors include the design (i.e., resolution) of the models, the lead time of the forecast or hindcast, and the optimal number of ensemble members. As an adequate treatment of each of these factors is clearly storm dependent, no overarching decision rules currently exist. Hopefully, as experience in making tropical cyclone attribution statements increases, these choices will become clearer.

The attribution method used here is a storyline approach to event attribution (*17*, *39*). As discussed in the recent work (*13*), this method compares two sets of simulations, the first being of the storm in the Actual world that is and the second being of the storm in a Counterfactual world that might have been had humans not altered the climate system. Attribution statements arising from this type of analysis are conditional on the changes to the atmospheric composition, the prescribed state of the Actual and Counterfactual ensemble ocean, the prescribed large-scale meteorological patterns, and the sensitivities of the model(s) used. Hence, only the effects of climate change local to the hurricane itself are included. These include structural changes to the storm driven by increased available sensible and latent heat energy. However, no statement can be made with this methodology about the changes in cyclogenesis rates or the probability of a Florence-like storm, as the specific large-scale atmosphere and ocean circulations that affect the genesis development of storms are prescribed. Despite these limitations, a clear picture of the response of intense tropical cyclones to global warming is emerging. An attributable increase in precipitation from recent intense tropical cyclones has already emerged for the storms analyzed so far by multiple independent author teams (see discussion above). This signal has emerged before the expected increases in storm intensity. As the climate continues to warm, it is expected that extreme tropical cyclone precipitation events and resulting inland flooding will become yet more frequent.

Extreme weather events, such as hurricanes and tropical cyclones, provide scientists with the opportunity to communicate the very real impacts of current climate change to the public. We demonstrated that a forecasted attribution analysis using a conditional attribution framework allows for credible communication to be made on the basis of sound scientific reasoning. Post-event expansion of the ensemble size and analysis demonstrated it to be reasonable, albeit with some quantitative modification to the best estimates and the opportunity to more rigorously evaluate the significance of the analysis. However, attribution statements, whether made before or after the event, must be made carefully and with sensitivity to the real-world circumstances. One particular suggestion is that forecasted attribution remarks should be made in conjunction with a statement to heed all warnings from the official forecast centers so as to not dilute the message of any clear and present danger. Attribution science offers a venue to engage the public with best estimates of the impact of climate change on the extreme weather events. The field has now advanced to the stage where we call on the scientific community to update the Intergovernmental Panel on Climate Change “detection and attribution best practices” guidance report (*40*) to include event attribution statements made either before or after the events.

## METHODS

The global atmospheric model CAM5 (*41*) was set up in a variable-resolution configuration (*42*) with 30 vertical levels and a base horizontal grid spacing of ~110 km, similar to conventional atmospheric general circulation models (AGCMs), and a refined region over the North Atlantic basin with a grid spacing of roughly 28 km. The grid is shown in fig. S1. Variable-resolution AGCMs reduced the boundary condition errors associated with regional climate models and used less computational resources compared with traditional globally high-resolution AGCMs, since fine grid spacing is not required over the entire domain. The model is initialized with atmospheric and ocean surface analyses from National Oceanic and Atmospheric Administration’s Global Forecast System. The land surface was initialized via iterative nudging. A forward digital filter was applied to the atmosphere during the first 6 hours of model integration to reduce gravity wave noise associated with remapping to the CAM5 grid. Specific details regarding the initialized configuration used in this study are described in (*31*).

Each ensemble simulation is run for 7 days, and model output is generated every 3 hours. For Hurricane Florence, the model was initialized every 12 hours starting 9 September at 12Z to 12 September at 00Z, inclusive. To account for model uncertainty in storm characteristics, a 10-member forecast ensemble was created by randomly perturbing three parameters (precipitation coefficient, c0_ocn; convective time scale, tau; and parcel fractional mass entrainment rate, dmpdz) in the deep convective parameterization based on results from tropical cyclone sensitivity studies in CAM5 (*43*). Note that the 11 September 00Z forecast period was expanded after storm to a 100-member ensemble to allow for a more thorough attribution analysis. These ensembles were taken to be the Actual hindcasts of Hurricane Florence. Storm trajectories from the individual simulations were computed by tracking the location of minimum sea level pressure using the TempestExtremes software package (*44*). The observed track, minimum sea level pressures, and maximum near-surface wind speeds for Hurricane Florence were obtained from the NHC’s operational best track product.

For the Counterfactual ensemble with the climate change signal removed, differences (or “deltas”) in the three-dimensional air temperature, three-dimensional specific humidity, and two-dimensional sea surface temperature were applied to the observed initial conditions to remove the effects of climate change (*13*). Data from the All-Hist and Nat-Hist CAM5 simulations under Climate of the 20th Century (C20C+) Detection and Attribution Project protocols, a coordinated international experiment specifically designed for event attribution, were used (portal.nersc.gov/c20c). The All-Hist simulations are a perturbed initial condition ensemble of lengthy Atmospheric Model Intercomparison Project—style simulations with prescribed sea surface temperature and sea ice concentrations derived from observations. The Nat-Hist simulations are a similar ensemble with the surface ocean and ice properties altered such that the attributable human components are removed. This removal was obtained by standard regression techniques from the Coupled Model Intercomparison Project Phase 5 ensemble (*45*). The attributable warming of the ocean surface off the coast of North Carolina is about 0.75°C in this protocol. Differences between global simulations driven by observed boundary conditions and simulations driven by conditions with the human-induced climate change signal removed were calculated for September over the 1996–2016 period and approximated the change in the large-scale environment attributable to climate change (*16*). In addition, the greenhouse gas concentrations, solar radiation conditions, ozone concentration, and aerosol concentrations are all set to their levels in the year 1850 for the Counterfactual ensemble. A second 100-member ensemble was created using these Counterfactual initial conditions. The same methodology was used to explore the climate change impacts on multiple recent hurricanes in the Weather Research and Forecasting model (*16*).

## Supplementary Material

http://advances.sciencemag.org/cgi/content/full/6/1/eaaw9253/DC1

Download PDF

Forecasted attribution of the human influence on Hurricane Florence

## SUPPLEMENTARY MATERIALS

Supplementary material for this article is available at http://advances.sciencemag.org/cgi/content/full/6/1/eaaw9253/DC1 Quantitative track error analysis Analysis of Hurricane Florence observed and forecasted rainfall Analysis of Hurricane Florence forecasted intensity Analysis of Hurricane Florence forecasted size Table S1. Comparison of track error. Fig. S1. CAM5 computational grid. Fig. S2. Actual ensemble rainfall. Fig. S3. Counterfactual ensemble rainfall. Fig. S4. Evolution of storm intensity and size. Reference (*46*)
