# Supplementary material for: Forecasted attribution of the human influence on Hurricane Florence
Source: Sci Adv. 2020 Jan 1;6(1):eaaw9253. doi: 10.1126/sciadv.aaw9253 (PMC6938700; doi:10.1126/sciadv.aaw9253)
Supplement: http://advances.sciencemag.org/cgi/content/full/6/1/eaaw9253/DC1 [file supp_6_1_eaaw9253__index.html]

Science Advances | Science AdvancesAAASSearchScience AdvancesMenu

## Supplementary Materials

**This PDF file includes:**

- Quantitative track error analysis
- Analysis of Hurricane Florence observed and forecasted rainfall
- Analysis of Hurricane Florence forecasted intensity
- Analysis of Hurricane Florence forecasted size
- Table S1. Comparison of track error.
- Fig. S1. CAM5 computational grid.
- Fig. S2. Actual ensemble rainfall.
- Fig. S3. Counterfactual ensemble rainfall.
- Fig. S4. Evolution of storm intensity and size.
- Reference (*46*)

Download PDF

**Files in this Data Supplement:**

- Adobe PDF - aaw9253\_SM.pdf
