# Supplementary material for: Forecasted attribution of the human influence on Hurricane Florence
Source: Sci Adv. 2020 Jan 1;6(1):eaaw9253. doi: 10.1126/sciadv.aaw9253 (PMC6938700; doi:10.1126/sciadv.aaw9253)
Supplement: Download PDF [file aaw9253_SM.pdf]

Science Advances
AAAS

advances.sciencemag.org/cgi/content/full/6/1/eaaw9253/DC1

Supplementary Materials for

Forecasted attribution of the human influence on Hurricane Florence

K. A. Reed*, A. M. Stansfield, M. F. Wehner, C. M. Zarzycki

*Corresponding author. Email: kevin.a.reed@stonybrook.edu

Published 1 January 2020, *Sci. Adv.* **6**, eaaw9253 (2020)
DOI: 10.1126/sciadv.aaw9253

This PDF file includes:

Quantitative track error analysis
Analysis of Hurricane Florence observed and forecasted rainfall
Analysis of Hurricane Florence forecasted intensity
Analysis of Hurricane Florence forecasted size
Table S1. Comparison of track error.
Fig. S1. CAM5 computational grid.
Fig. S2. Actual ensemble rainfall.
Fig. S3. Counterfactual ensemble rainfall.
Fig. S4. Evolution of storm intensity and size.
Reference (*46*)

Quantitative track error analysis

A simple track error analysis of the Actual ensemble was undertaken to verify skillful prediction of Hurricane Florence by CAM5. Table S1 shows the "equal event" track error (in nautical miles) for the CAM5 ensemble, the National Centers for Environmental Prediction (NCEP) GFS ensemble (GEFS), and the NHC official forecasts. Statistics are calculated using the Model Evaluation Tools (MET) package.[1]

Table S1. Comparison of track error. Table of average hindcast track error as a function of lead time for the CAM5 ensembles (CAM5), the NCEP GFS ensemble (GEFS), and official National Hurricane Forecast (Official). Six initialization times are considered (Sept. 9 12Z through Sept. 12 00Z, inclusive). CAM5 tracks are generated using TempestExtremes; GEFS and Official statistics are derived from NHC a-deck files.

Lead (hours)	CAM5 (nautical miles)	GEFS (nautical miles)	Official (nautical miles)
24	40.5	14.8	13.7
48	44.0	33.5	19.6
72	37.0	58.0	31.8
96	70.4	126.8	75.4
120	72.6	226.9	117.5

Results show that the CAM5 ensemble has higher track error at small lead times, likely due to the 28 km grid being coarser than that of current operational products, thereby limiting the minimum resolvable spatial differences of the storm center. At longer lead times of >= 72 hours, the model performs skillfully, registering track errors that are smaller than both GEFS and NHC forecasts out to 5 days (120 hours). It should be emphasized that this sample size is far too small to make definitive statements about general CAM5 performance, although these results qualitatively agree with previous work showing that CAM5 initialized with realistic conditions can produce tropical cyclone hindcast skill similar to operational weather forecasting products *(31)*.

Analysis of Hurricane Florence observed and forecasted rainfall

To isolate the precipitation produced by Hurricane Florence, the total rainfall at all overland model grid points within 500 km of the simulated storm's landfall point is extracted at each 3-hour interval. The extracted precipitation is then accumulated in time

1 Available at https://dtcenter.org/met/users/

over the modeled storms lifetime to create an accumulated precipitation product. The observed precipitation is from the National Weather Service (NWS) Advanced Hydrologic Prediction Service's 6-hourly quantitative precipitation estimates and is conservatively remapped to the CAM5 model grid as shown in Fig. 2. A similar rainfall analysis is performed for all Actual and Counterfactual ensemble analysis for 100-member Sept. 11 00Z ensemble simulations and a subset of these ensembles is shown in fig. S2 and S3.

Analysis of Hurricane Florence forecasted intensity

One measure of the intensity of a tropical cyclone is the maximum near-surface wind speed. The time evolution of the maximum near-surface wind speed calculated from the 2D wind field for the Actual and Counterfactual ensembles for Hurricane Florence is shown in fig. S4. To compare the maximum near-surface winds from the model output to the observations, the winds at the lowest model level (~64 m) were used to estimate the model winds at 10 m using the logarithmic model of boundary layer. An alternate measure of intensity is the minimum surface pressure of the tropical cyclone. The time evolution of the minimum surface pressure for the Actual and Counterfactual ensembles is shown in fig. S4. While both measures of intensity indicate that the Counterfactual storm is weaker than the observed storm, the differences in ensemble median intensities are minimal, with overlap of the interquartile range. This intensity signal (or lack thereof) is consistent with attribution studies for recent hurricanes *(16)*. It is worth noting that when compared to observations, the Actual ensemble produces a somewhat stronger storm in the time leading up to landfall (after an initial spin up in the first 24 hours).

Analysis of Hurricane Florence forecasted size

The storm outer size metric used for this analysis is the radius of the azimuthally-averaged 8 m/s azimuthal wind (*r8*). This radius is calculated by first splitting the wind field into radial and azimuthal components based on the storm center location calculated by TempestExtremes. Next the azimuthal wind components are split into bins and averaged azimuthally around the storm center, which creates a radial wind profile of the storm. The distance from the storm center where the wind is equal to 8 m/s is recorded as *r8*. More details on the method behind calculating *r8* is described in previous work *(46)*. The time evolution of *r8* for Hurricane Florence for the Actual and Counterfactual ensembles is shown in fig. S4.

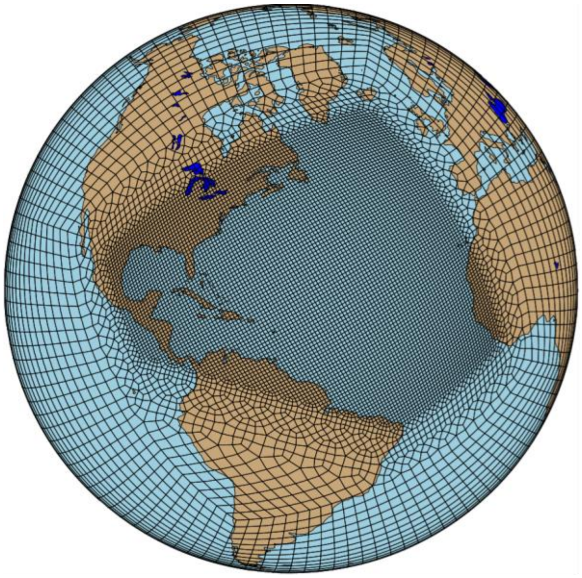

Fig. S1. CAM5 computational grid. CAM5 variable-resolution grid used in this study highlighting regional refinement (28 km) over the North Atlantic ocean basin. Note that each element outlined here contains an unplotted 3x3 matrix of collocation grid cells that defines to final model grid spacing.

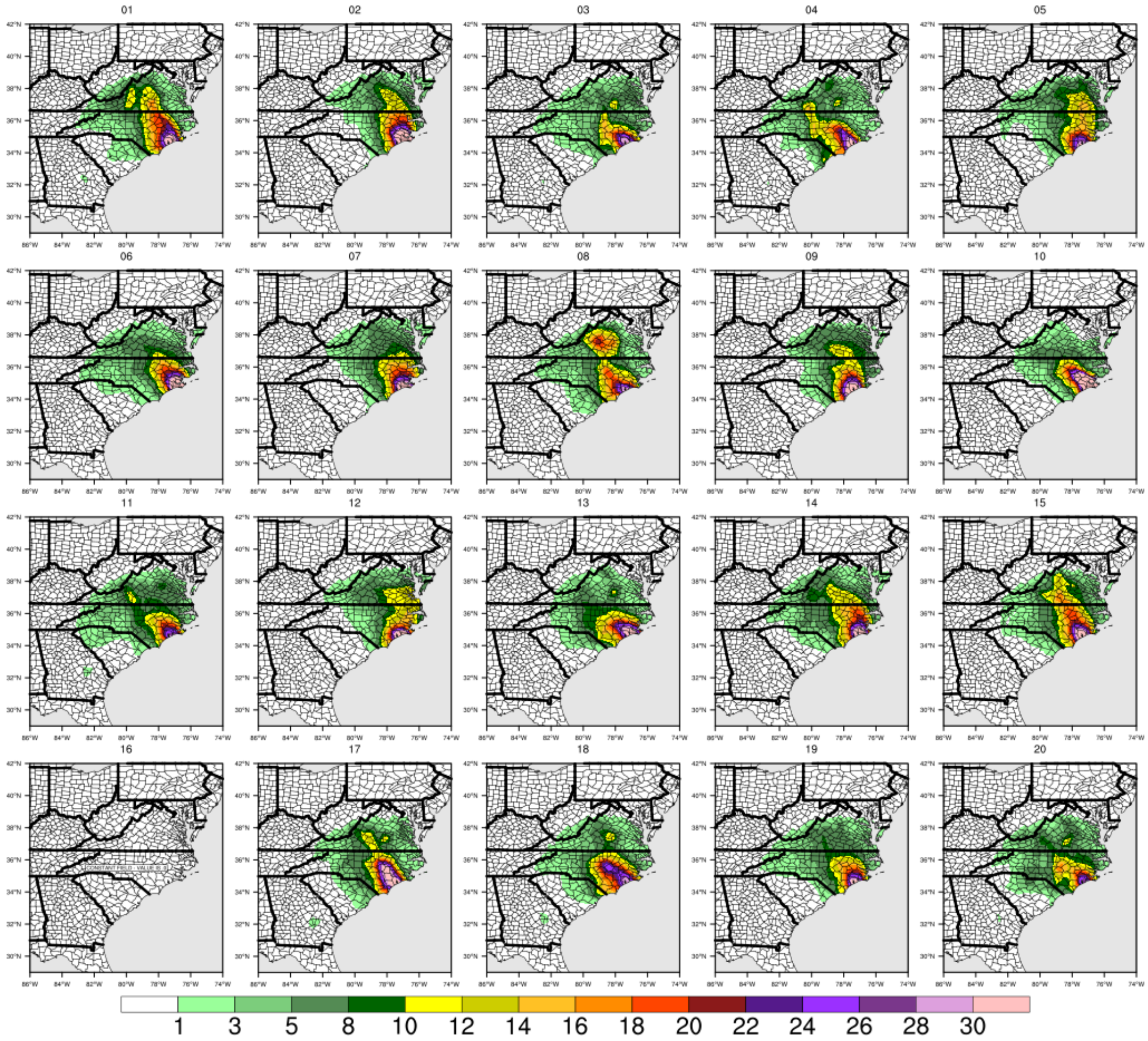

Fig. S2. Actual ensemble rainfall. Total accumulated precipitation (inches) within 500 km of the model storm's landfall location over the entire forecast period for the first 20 ensemble members for the Sept. 11 00Z initialization time for the Actual ensemble.

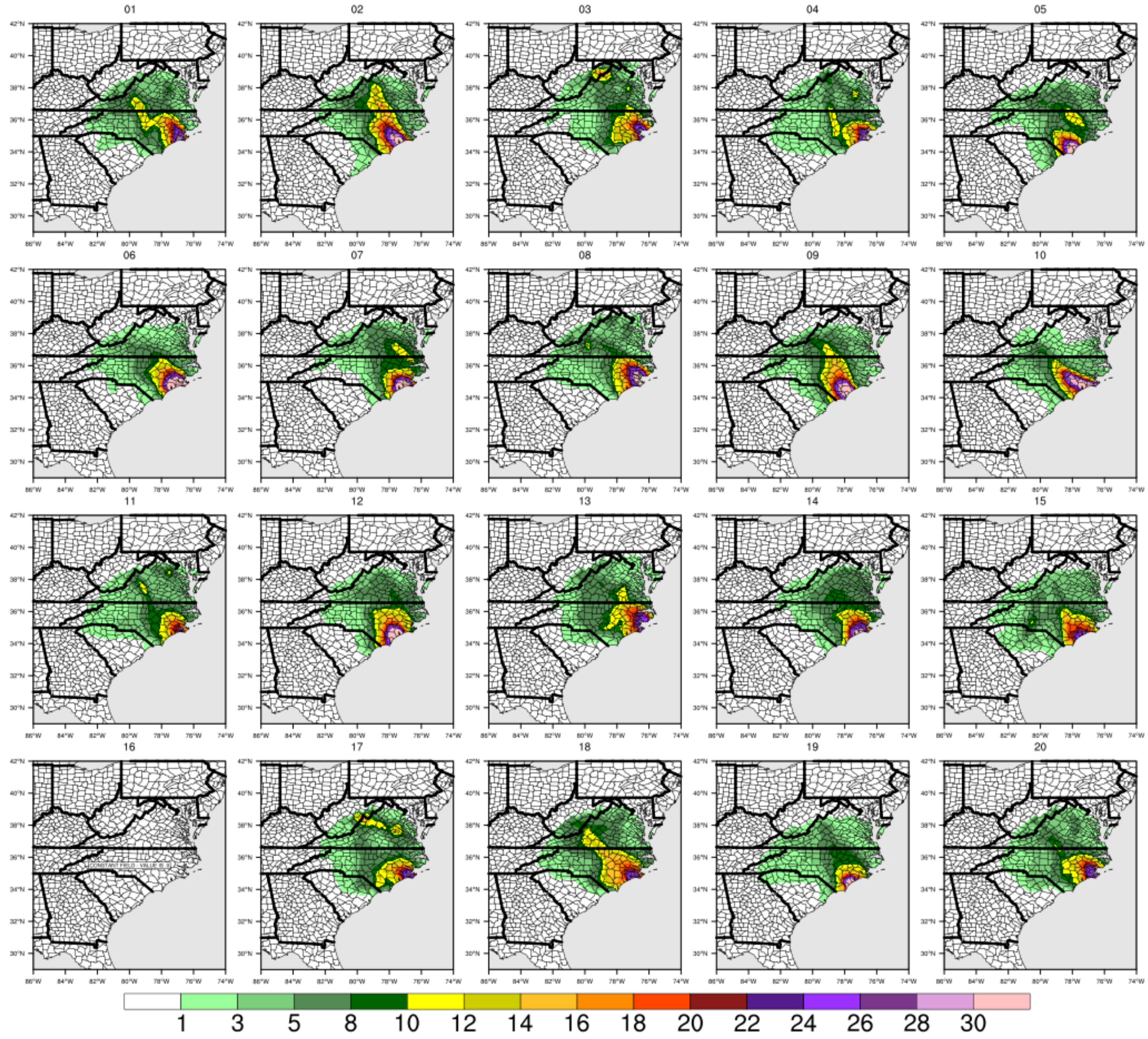

Fig. S3. Counterfactual ensemble rainfall. Total accumulated precipitation (inches) within 500 km of the model storm's landfall location over the entire forecast period for the first 20 ensemble members for the Sept. 11 00Z initialization time for the Counterfactual ensemble.

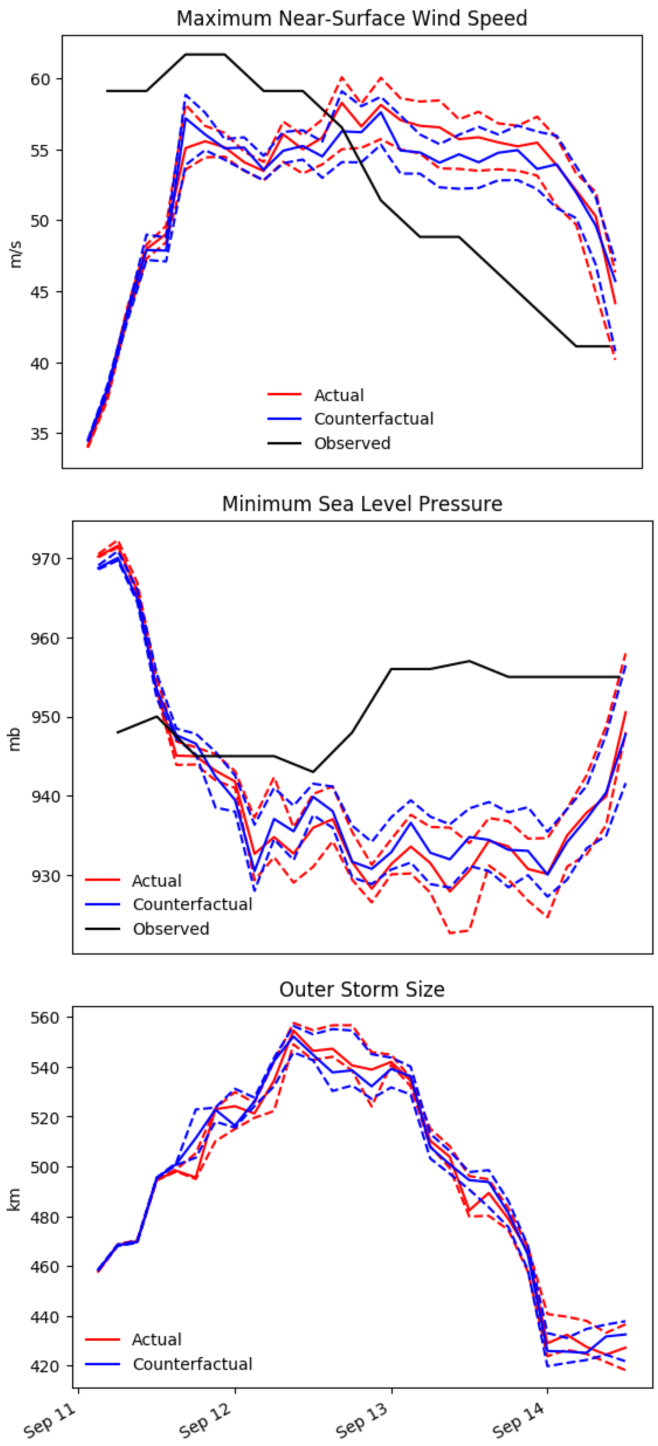

Fig. S4. Evolution of storm intensity and size. Time series of the ensemble median (solid) and interquartile range (dotted) of the maximum near-surface wind speed (top), minimum sea level pressure (middle), and outer storm size *r8* (bottom) for the Actual (red) and Counterfactual (blue) Sept. 11 00Z ensembles. Only the 96 ensemble members that make landfall within 200 km of the observed landfall location are included. The time series ends the time step before the ensemble median landfall time for each ensemble. NWS observations (black) for wind speed and minimum sea level pressure are also provided.
